# The effects of performing integrated compared to isolated core exercises

**DOI:** 10.1371/journal.pone.0212216

**Published:** 2019-02-27

**Authors:** Atle Hole Saeterbakken, Ajit Chaudhari, Roland van den Tillaar, Vidar Andersen

**Affiliations:** 1 Western Norway University, Faculty of Teacher Education, Culture and Sport, Department of Sport, Food and Natural Sciences, Sogndal, Sogn og Fjordane, Norway; 2 The Ohio State University, Physical Therapy, School of Health & Rehabilitation Sciences, Columbus, Ohio, United States of America; 3 Nord University, Department of Sport Science and Physical Education, Levanger, Norway; Hochschule Trier, GERMANY

## Abstract

Integrated exercises that mimic daily tasks are generally preferred for improving performance and the later stages of rehabilitation, but it is unknown whether integrated core exercises are better than isolated core exercises at improving muscle activation for hypertrophy. The aim of the study was to compare the electromyographic (EMG) activity in rectus abdominis, oblique externus, and erector spinae while performing three conditions of integrated core exercises (lunges) with three isolated core exercises (prone bridge, side bridge and back extension). The three conditions of lunges were: on a stable surface, unstable surface and with external resistance to the trunk using an elastic band. The external resistance was measured with a force cell and peaked at 75N. After one familiarization session, all exercises were performed in one experimental session in randomized order. The isolated core exercises were performed in 20 seconds and the time performing the five repetitions with lunges was matched (20 seconds). Significantly greater peak normalized EMG activity were observed in the isolated core exercises compared to the three integrated core exercises (P<0.001) with two exceptions. For the oblique externus, the isolated core exercise was only greater than the stable lunge. Lunges with elastic bands only demonstrated greater peak erector spinae activation compared the other lunge conditions. Comparing the mean EMG activity between the isolated and three integrated exercises, greater muscle activations were observed performing the isolated exercises (P<0.001). Unstable lunges did not increase the peak or mean core muscle activations. In conclusion, mean and peak EMG activity performing the isolated exercises were in general greater than the three condition of lunges. Based on these results, we recommend using isolated core exercises when the primary goal is to improve muscle activation and elicit hypertrophy, but integrated exercises once adequate initial hypertrophy is achieved.

## Introduction

Adaptations in the musculoskeletal system to enhance performance are specific to the training the system is exposed to. To enhance performance or rehabilitation, training programs should target specific muscular deficits and establish optimal exercises to target specific muscular performance deficits. There are countless exercises that target the core muscles to promote and improve athletic performance, general health and prevent low back pain [1–5]. Most of these studies have compared muscle activation of core using exercises trying to isolate specific core muscles which do not mimic core muscle activation in daily living activities. It`s generally accepted that improved core stability may provide a foundation for greater force production [6–8]. The use of unstable surfaces (i.e. BOSU balls, wobbler boards, Swiss ball, slings) is the most common way to increase the stability requirement [2, 9–12]. Performing exercises on an unstable surface has increased the proprioceptive demands and resulted in greater muscle activation than a stable surface [2, 4, 13–16]. On the other side, an unstable surface (i.e. BOSU balls) has a much greater stability requirement than typically required in daily living activities.

Core muscle activation has been compared in different conditions of strength training exercises. For example, performing exercises in standing instead of seated/supine [17, 18] and unilateral instead of bilateral [11, 16, 19–21] increased the core muscle activation. In addition, several studies have compared the core muscle activation performing different isometric core exercises targeting isolated muscles (i.e. prone bridge, side-bridge) [15, 16, 22–24]. Lately, studies have compared isolated exercises with integrated exercises (i.e. multi joint exercises as squats) and demonstrated greater or similar muscle activation with integrated exercises [25–28]. For example, high-intensity squats and deadlifts (>70% of 1RM) caused similar or greater core muscle activation compared with core-isolating exercises such as the side-bridge and prone bridge [29–31].

The lunge is a functional weight bearing task and is commonly prescribed as a therapeutic exercise to strengthen the lower extremity and simulate activities of daily living (i.e. jogging, stairs climbing or jumping) [32]. The lunge is performed unilaterally and in a standing position which increases the stability requirement of the core [17, 18, 20] and integrates the core muscles in the kinetic chain with the lower extremity muscles. To our knowledge, only Ekstrom et al. [33] have compared the lunge exercise with traditional core exercises. They reported greater longissimus thoracis, multifidus spinae, oblique externus, and rectus abdominis activity when performing the side- and prone-bridges compared to lunges. However, the lunges were performed with a 5-second hold at the point with maximal knee flexion and thereby reduced the specificity towards daily living tasks. Furthermore, muscle activation was analyzed as the mean of one repetition. This approach may mask the natural variation in the activation patterns of performing a lunge as the exercise changes over its multiple phases. When including different conditions of the lunge (stable surface, unstable surface and with external loads to the core) a more detailed view could be established of the core muscle activation during this exercise. Furthermore, comparing lunges to isometric core exercises would give more insight into the differences between these types of exercises and thereby help trainers, therapists and athletes in planning training for core strength that is more effective and more closely matched to daily tasks and movements.

To the best of our knowledge, no previous studies have compared isometric core exercises frequently used in rehabilitation with integrated dynamic core exercises frequently used in prevention of low back pain. Therefore, the aim of the study was to compare the core muscle activation in three different conditions of the lunge (stable surface, unstable surface and with external loads to the core) with traditional isometric core exercises (prone bridge, side bridge, and back extension). We hypothesized similar core muscles activation between lunges with external loads and the isometric core exercises, but greater than the lunges on stable and unstable surfaces.

## Methods

### Design

A within-subjects repeated measure design was used to examine the differences in core muscle activation performing three variations of integrated dynamic core exercises (lunges on a stable surface, unstable surface and with resistance on a stable surface) compared to isolated isometric core exercises (prone bridge, side bridge, and back extension). To add external resistance, while performing the lunges, an elastic band was placed over the chest connected anterior (stressing the erector spinae), lateral (stressing the oblique externus) or posterior (stressing the rectus abdominis). A total of five sets of lunges were performed by each participant. All exercises were performed in one session in the experimental test. Only the prime muscle in the lunges with external resistance were compared with the prime muscle performing the isometric exercises.

### Participants

Twenty-one healthy recreational trained females (age = 21.6 ± 1.7 years, stature = 1.67 ± 0.05 m, body mass = 66.5 ± 8.3 kg) participated in this study. All of the participants had resistance training experience (4.2 ± 2.7 years) but were not competitive power or weight lifters. The participants were accustomed to the exercises, but were instructed to refrain from any additional resistance or core training 48 hours before the test. Prior to the study, each subject was informed of the testing procedures and possible risks, and a written consent was obtained from each participant.

### Ethics statement

The study was approved by the Norwegian Centre for Research Data before the start of the study. The study conformed with the latest revision of the Declaration of Helsinki, the ethical guidelines at the Sogn og Fjordane University College (Norway) and Norwegian laws and regulations. Participants were informed (both in writing and orally) about all testing and training procedures and gave written informed consent to participate prior to entering the study. In addition, the participant gave written informed consent (in accordance with PLOS consent guidelines) for the images to be reproduced in this manuscript. The individual in this manuscript has given written informed consent (as outlined in PLOS consent form) to publish these case details.

### Testing procedures

**Fig 1:**
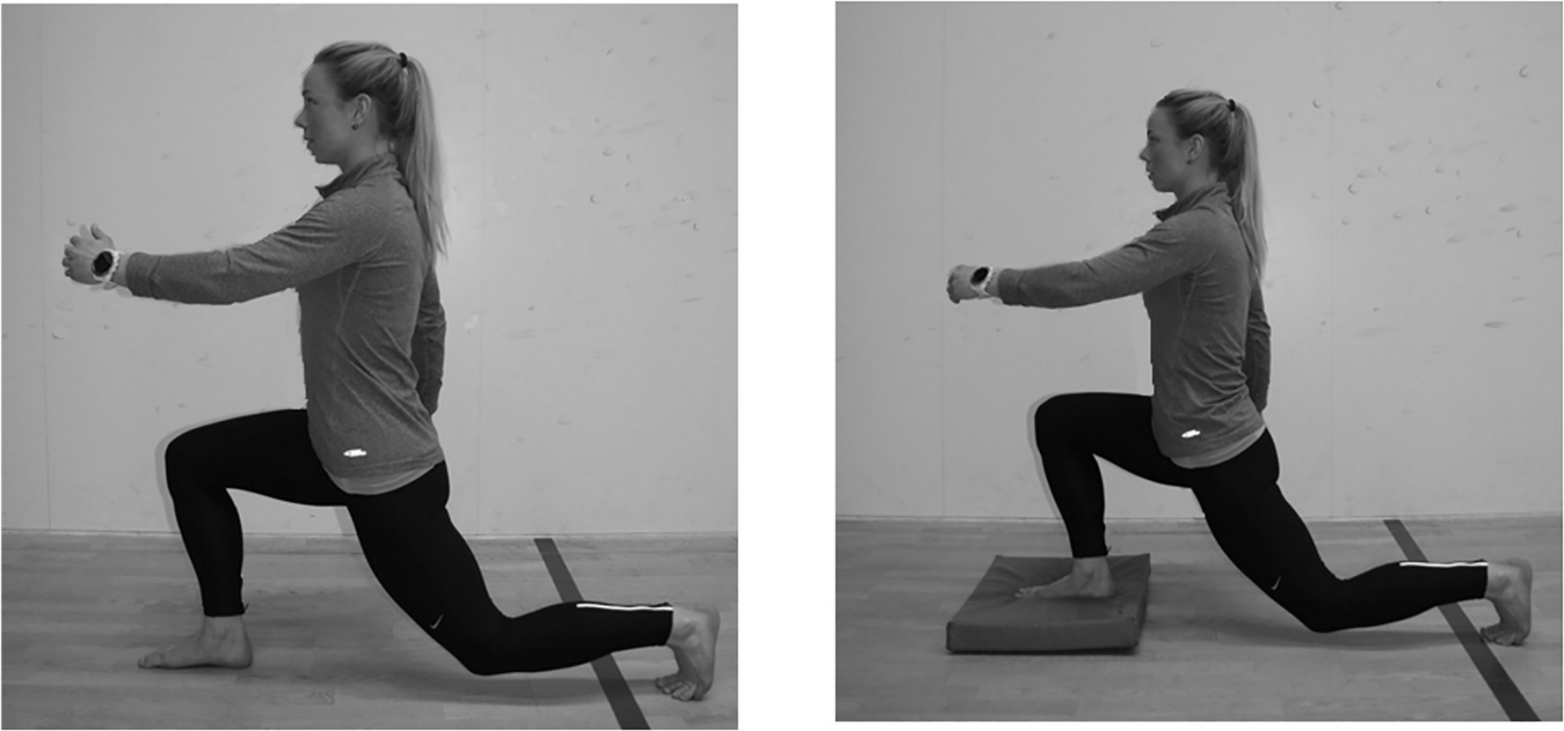
The lunge on stable surface or on an unstable surface.

Participants performed all exercises in a randomized order in each of two sessions, the familiarization session (3–10 days prior) and the testing session. On the familiarization session, the distance between the trochanter major and the ground was measured. The same length was measured on the floor and used to determine the distance of the lunges. The participants placed their heel on one marker and had to step passed the next with their heel to make a successful repetition. The lunges were performed barefoot with the same starting position (standing straight) with a natural sway in the back (see Fig 1). The lunges were performed barefoot to avoid differences in ankle support from the participants`different shoes. Shoes with better ankle support enable better balance, stability and control than shoe with less support [34–36].

Before starting the lunges, the participants were asked to stand straight and look straight ahead with arms along the side. The participants were then instructed to maintain upright torso and back posture while performing the lunges, but were instructed the use contralateral arm to mimic a stride. The same back posture had to be maintained and only the preferred foot was tested. The eccentric movement stopped when the knee of the preferred foot was over the toes and the opposite knee was close, but not touching, the floor (see Fig 1). All participants conducted six repetitions of each type of lunge where the last five repetitions were used for further analyses. A repetition lasted in four seconds (two seconds in the eccentric and concentric phase) and a metronome (60 bpm) was used to control the speed. The arms were allowed to swing as in a normal walking stride. The lines between the iliac crests and between the acromions on the shoulders had to be horizontal during the lunges. If the participants lost balance, the exercise was aborted and a new attempt to complete six repetitions was made. Each set of six lunges were separated with a two-minute rest break. The participants used between 1–2 attempts to complete the tests.

**Fig 2:**
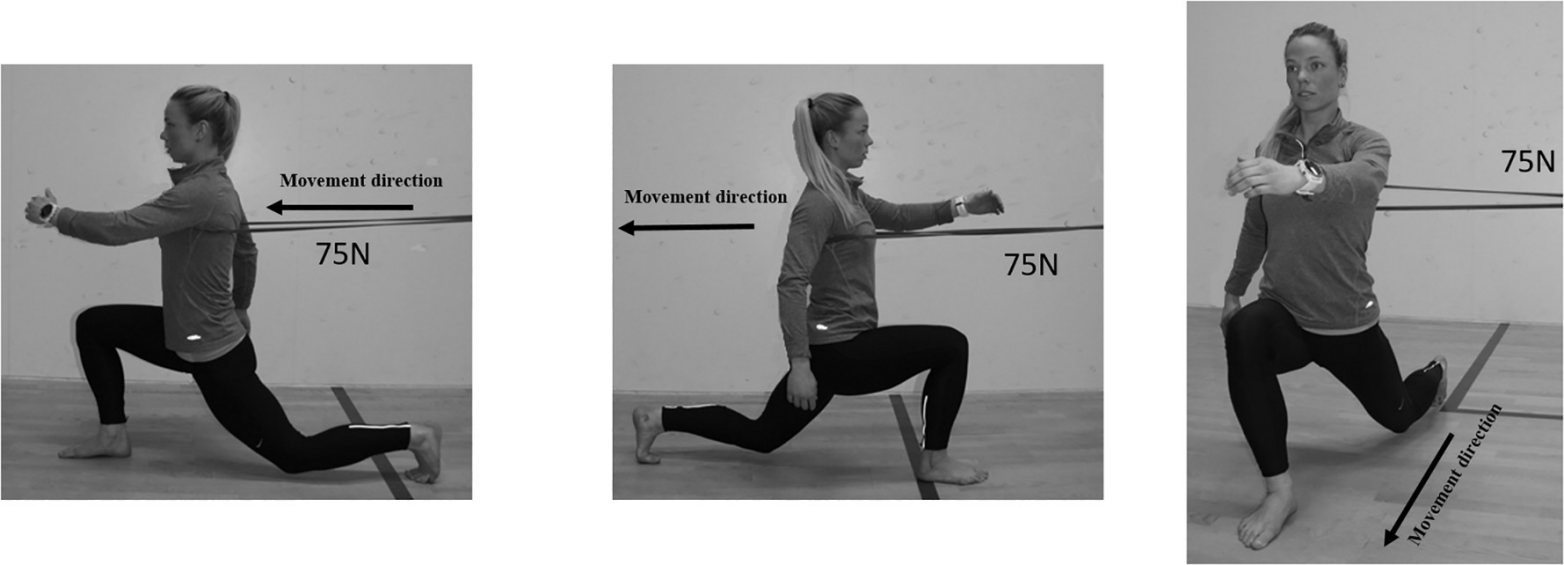
The lunge with external loading in a forward lunge, backward lunge and forward lunge with elastic band from the side.

Three variations of the lunges were performed as exercises meant to integrate the core muscles. The variations were lunges on 1) a stable surface, 2) unstable surface or 3) stable surface with an external, horizontal resistance. The variations of the lunges were performed without any additional weights or vertical resistance. When performing lunges on an unstable surface (Fig 1), the foot stepping forward had to land on a balance pad (Airex balance pad elite, Fysiopartner AS, Norway). Performing the lunges on a stable surface, the approach was identical as the unstable surface, but without the balance pad (Fig 1). The lunges with external resistance were performed in three series with the added external resistance attached to the upper body in three different directions (anterior, posterior and lateral) to emphasize the different core muscles. The elastic band was attached anterior of the participants when the participants performed a backward lunge. The participants had to activate the erector spinae more to maintain a natural sway of the lower back (Fig 2), while the rectus abdominis had to be activated more when elastic band was attached posteriorly when the participants performed a forward lunge (Fig 2). Attaching the elastic band lateral of the participants while performing a forward lunge, the oblique muscles had to be activated more on the contralateral side (Fig 2). The elastic band was therefore placed on the left side if the dominant foot of the participants was right. The external resistance from the elastic band was measured with a force cell (Ergotest Technology AS, Langesund, Norway) attached to the elastic band (Fig 2). The average (minimum and maximum) resistance performing the repetitions with the elastic bands placed lateral, anterior and posterior were 71.6±0.8N (69.2±1.5N and 81.7±1.5N), 81.0±1.8N (73.3±1.5N and 88.0±1.6N) and 52.9±0.9N (37.1±1.3N and 74.0±1.8N).

**Fig 3:**
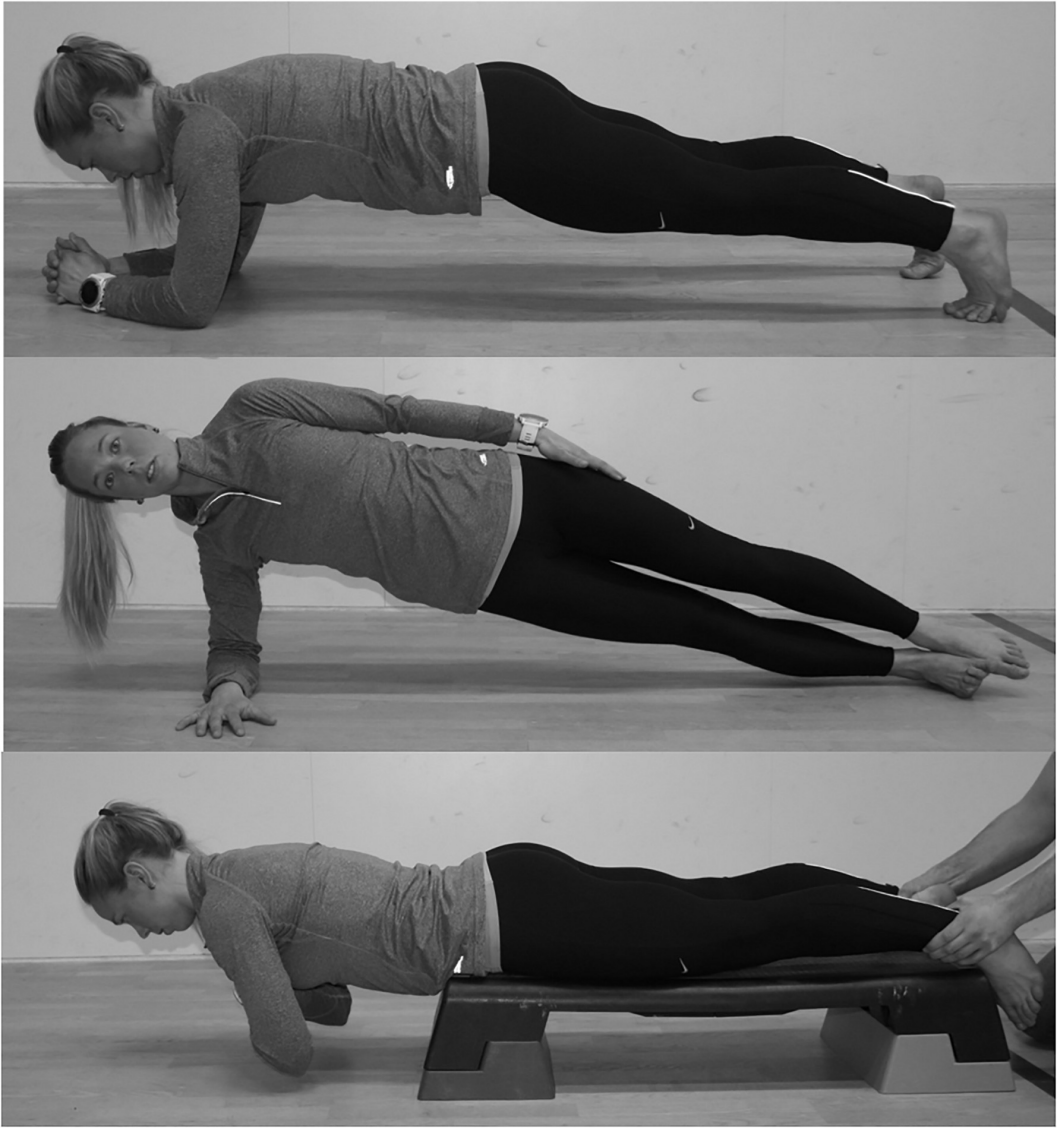
The three isolated core exercises prone bridge, side-bridge and back-extension.

Three different exercises were used to challenge each core muscle in an isolated state. The exercises were the prone bridge (rectus abdominis), side bridge (oblique externus), and back extension (erector spinae) (Fig 3). All exercises were performed in 20 seconds to match the working time of the lunges [29]. All three exercises were performed with a neutral alignment in the spine and pelvis [37]. The prone bridge was performed with 90 degrees of elbow and shoulder flexion and 30 degrees of ankle plantarflexion (Fig 3). The distance between the toes and elbow was measured during the familiarization session and repeated in the experimental session [37].

To perform the side bridge, the participants placed their upper foot anterior to the lower. The exercise was performed on the forearm with a straight line through the body (Fig 3). The distance between the forearm and lower foot was measured in the familiarization session and repeated in the experimental session.

The Biering-Sorensen test was used to isolate the erector spinae in the back extension [21, 38]. The participants lay prone on a table with a horizontal position (Fig 3). The tip of the iliac crest rested on the edge of the table with the arms folded across the chest. A person held the participant's ankles.

### Measurements

Surface EMG electrodes were positioned on the rectus abdominis (3 cm lateral to the umbilicus), the oblique externus (approximately 15 cm from the umbilicus), and the erector spinae (at L1 and 3 cm lateral to the spinous process) (5, 17, 25). Prior to the placement of the gel-coated self-adhesive electrodes (Dri-Stick Silver Circular sEMG Electrodes AE-131, NeuroDyne Medical, USA), the skin was shaved, washed with alcohol, and abraded (17). The electrodes (contact diameter = 11 mm, centre-to-centre distance = 20 mm) were placed on the core contralateral to the side of the dominant leg (5, 26). A commercial EMG recording system was used to measure the EMG activation (MuscleLab 4020e, Ergotest Technology AS, Langesund, Norway). To minimize the noise induced from external sources through the signal cables, the EMG raw signal was amplified and filtered using a preamplifier located as close as possible to the pickup point. The preamplifier had a common mode rejection ratio of 100 dB. The EMG raw signal was then bandpass filtered (fourth-order Butterworth filter) with cut-off frequencies of 8 Hz and 600 Hz. The filtered EMG signals were converted to RMS signals using a hardware circuit network (frequency response = 0–600 kHz, averaging constant = 100 ms, total error = ± 0.5%). Finally, the RMS-converted signal was sampled at 100 Hz using a 16-bit A/D converter (AD637). Commercial software (MuscleLab V8.13, Ergotest Technology AS, Langesund, Norway) was used to analyze the stored EMG data.

**Fig 4:**
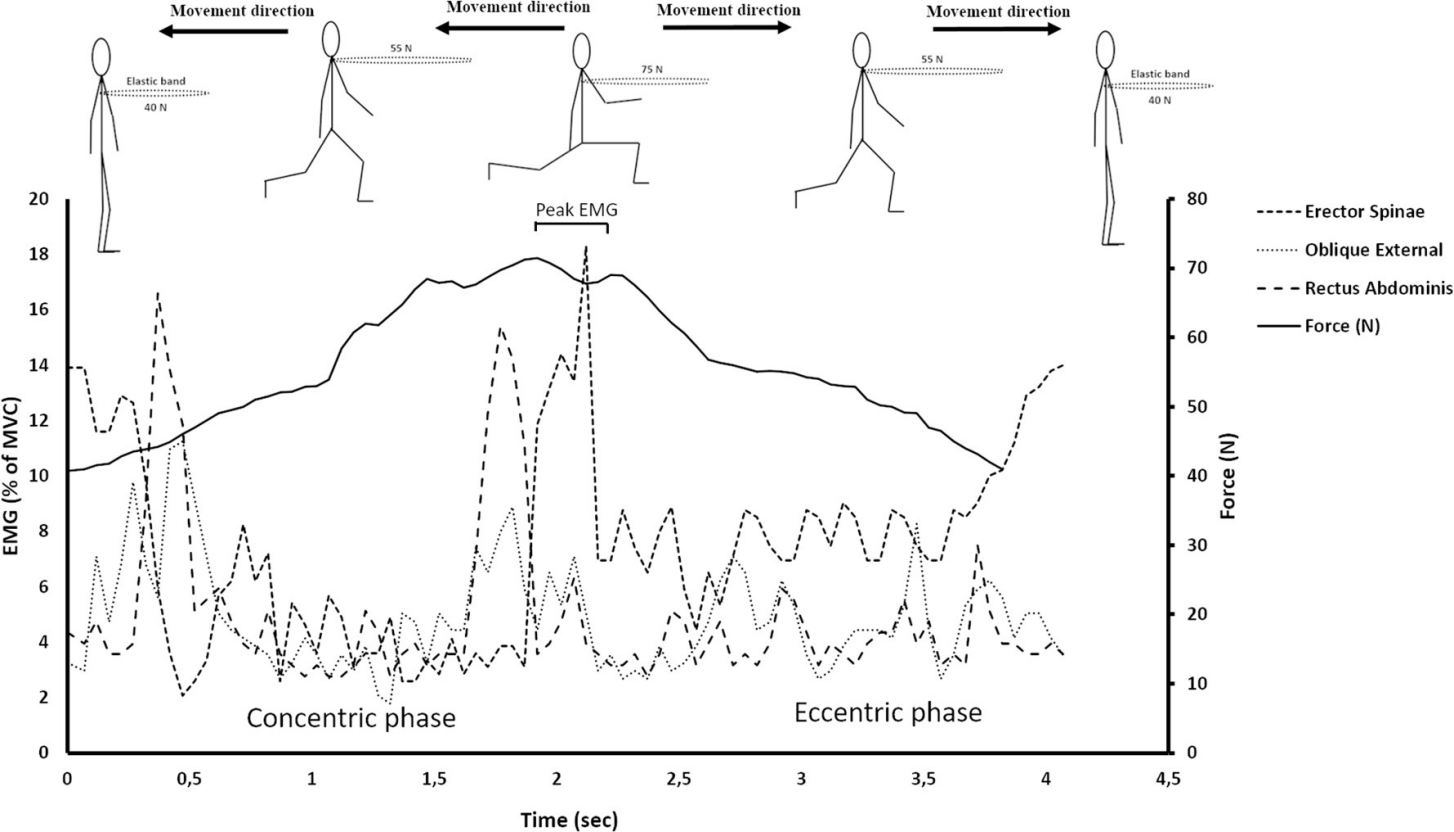
A typical example of the variation of core muscle activation performing a backward lunge.

A linear encoder (100Hz sampling frequency, synchronized with EMG; ET-Enc-02, Ergotest Technology AS, Langesund, Norway) was attached to the participants’ hip to identify the beginning and end of each repetition. The linear encoder was synchronized with the EMG measurements with the Musclelab 4020e hardware (Ergotest Technology AS, Langesund, Norway). The beginning of the second repetition and the end of the sixth repetition was identified for each of the three lunge conditions (5 repetition lasting 4 seconds = 20 seconds period). A similar approach was used for the isolated core exercises. The participants established the starting position and when a correct position was established, a test-leader pulled the encoder slightly to mark the data with a little spike to identify the start of the exercise. The participants than maintained the position and after 20 seconds (identified by a stop watch), the test-leader moved the encoder again to mark the end of exercise. The mean EMG RMS activities over the 20 second period were calculated for each exercise and used for further analyses. In addition, the highest value of the 500-ms running average of the EMG activity was identified as the peak EMG (see Fig 4) for each repetition of the three conditions of lunges. From the five repetitions, the mean peak EMG activity of these five repetitions was used for further analyses. Finally, all EMG data was normalized to a 5-second maximal voluntary isometric contraction (MVC). The average value over the 3 seconds with the greatest EMG activity was used as the MVC. The sit-ups (90-degree hip and knee flexion), back extension (prone on the floor) and sit-ups with a rotation in the upper body were used to measure MVC and are described in detail elsewhere [27, 33]. Manual resistance was added to each exercise to elicit MVC [29].

### Statistical analysis

To assess differences in the mean and peak muscle activity between the core exercises (prone bridge, side bridge and back extension) and the three conditions of lunge (on a stable surface, unstable surface and with external loading), mixed-effects models were created for the normalized average activation and normalized peak activation of each muscle with subject as a random effect and condition as a fixed effect. Tukey’s HSD tests were used identify significant pairwise differences between conditions. An effect size of 0.2 was considered small, whereas 0.5 and 0.8 were considered medium and large, respectively. JMP 10 (SAS Institute, Cary, NC, USA) was used for the statistical analyses. P ≤ 0.05 was considered statistically significant.

## Results

Summary results are presented in Table 1 as least squares means ± 95% confidence intervals and with estimated Cohen’s d effect size (ES) when comparing the exercises.

**Table 1:** A summary of the results comparing the exercises.

Muscle	Condition	Least Square Mean (%MVC*)	95% CI*	Difference from Stable	SE* of Difference from Stable	Estimated Cohen's d with n = 20
Erector Spinae	Isolated	39,5	[33.6,45.4]	21,1	3,1	1,52
	Elastic	28,3	[22,34.6]	9,9	3,2	0,69
	Unstable	19,5	[13.4,25.7]	1,2	3,2	0,08
	Stable	18,3	[12.3,24.4]	-	-	-
Obliquus Externus	Isolated	44,3	[32.3,56.3]	19,8	7,3	0,61
	Elastic	38,5	[25.6,51.4]	14,1	7,5	0,42
	Unstable	26,5	[13.6,39.4]	2,0	7,5	0,06
	Stable	24,5	[11.9,37.1]	-	-	-
Rectus Abdominis	Isolated	45,0	[35.5,54.5]	16,9	5,3	0,72
	Elastic	29,6	[19.7,39.6]	1,5	5,3	0,06
	Unstable	29,0	[19.3,38.8]	0,9	5,3	0,04
	Stable	28,1	[18.4,37.9]	-	-	-
Erector Spinae	Isolated	39,7	[35.3,44]	23,5	1,9	2,73
	Elastic	20,8	[16.4,25.1]	4,6	1,9	0,53
	Unstable	17,0	[12.6,21.3]	0,8	1,9	0,09
	Stable	16,2	[11.9,20.6]	-	-	-
Obliquus Externus	Isolated	44,3	[33.5,55.1]	28,3	6,2	1,02
	Elastic	24,6	[13.8,35.4]	8,6	6,2	0,31
	Unstable	21,7	[10.9,32.5]	5,7	6,2	0,21
	Stable	16,0	[5.2,26.7]	-	-	-
Rectus Abdominis	Isolated	45,0	[39.2,50.9]	28,8	3,3	1,95
	Elastic	15,7	[9.9,21.4]	-0,5	3,3	-0,04
	Unstable	17,2	[11.5,22.9]	1,0	3,3	0,07
	Stable	16,2	[10.4,21.9]	-	-	-

*MVC = Maximal Voluntary Contraction, CI = Confidence Interval, SE = Standard Error.

**Fig 5:**
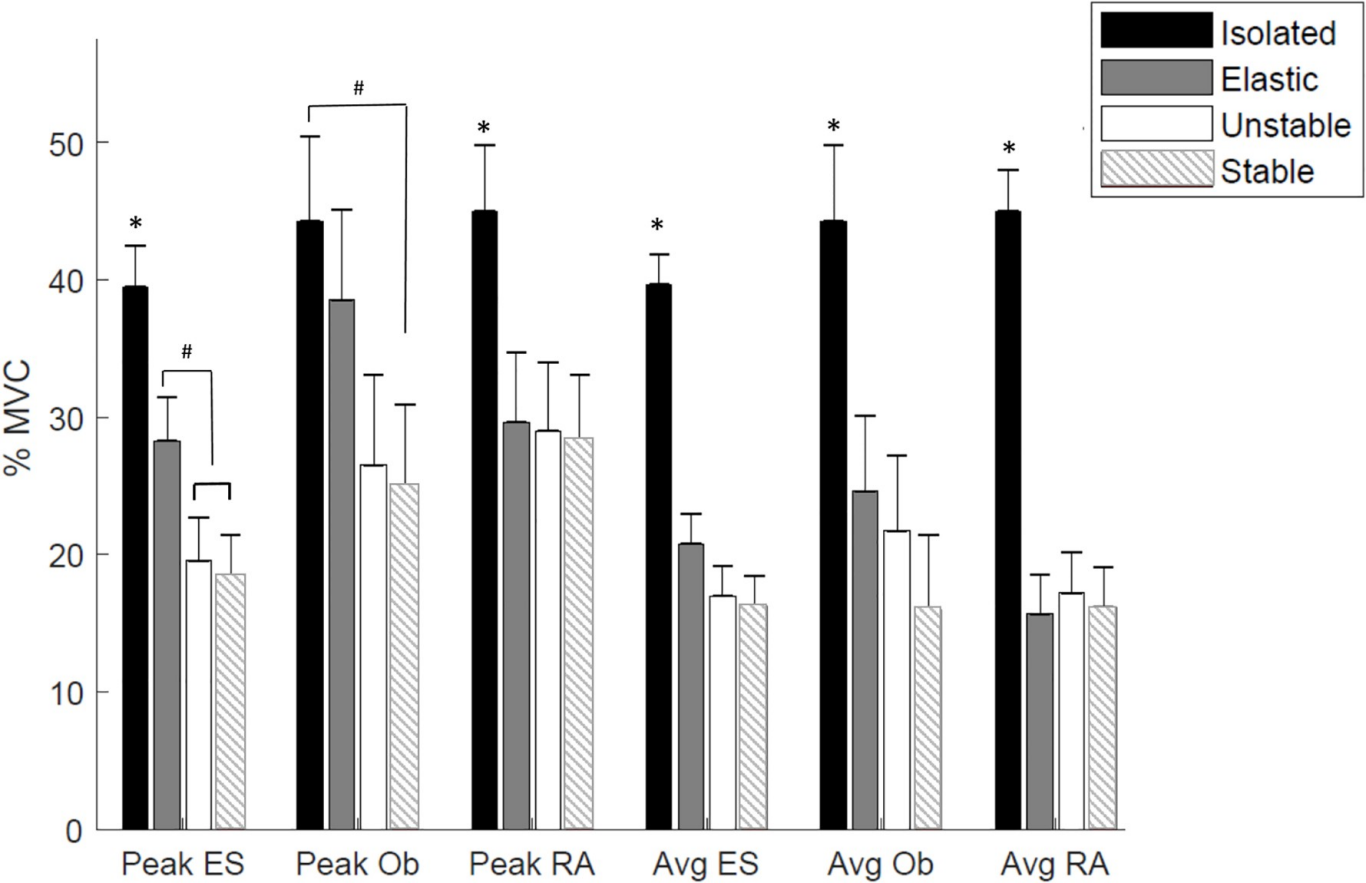
The normalized peak and average muscle activity (mean ±SD) in the different exercises.

Significant differences were observed in peak normalized EMG activity in rectus abdominis, oblique externus and erector spinae between exercises (all P<0.0001, Table 1) using the mixed effects model (Fig 5). The subsequent pairwise comparisons showed that for erector spinae and rectus abdominis, the isolated exercises resulted in significantly higher peak activity than all lunge conditions. For oblique externus, the only significant different in peak activity was between isolated and the stable lunge. For peak erector spinae activation, the lunge with elastic bands was also significantly higher than the unstable or stable lunges.

When comparing the mean EMG activity in rectus abdominis, oblique externus and erector spinae during the different core exercises (Table 1), again the fixed effect of condition was statistically significant (all P<0.0002). Subsequent pairwise comparison demonstrated that for each muscle, the isolated exercise elicited significantly higher mean EMG activity than all lunge conditions. However, no significant differences were observed in mean EMG activity between lunge conditions (Fig 5).

## Discussion

The main findings of this study were that the isolated core exercises demonstrated greater average and peak core muscle activation compared with the three lunge conditions (integrated core exercises) in most conditions. Differences between the lunge with the elastic band and the other lunges were small to moderate for the erector spinae, small to moderate for the oblique externus, and minimal for the rectus abdominis. The unstable lunge did not appear to provide any benefit over the stable lunge in these muscle activations.

As hypothesized, greater average core activation were observed between the isolated core exercises and the three integrated core exercises (lunges). Greater core muscle activation during isolated core exercises when analyzing the mean activation of the different lunge conditions, may be a result of different torque acting upon the core [18, 20]. All isolated core exercises were performed horizontal with a greater moment arm of the core than performing the lunge in a vertical position of the core. Further, the gravity will cause greater loading of the center of the body (core) in a horizontal position than a vertical. Both factors may explain the lower muscle activation when performing the different lunge conditions. This speculation is consistent with the findings of Comfort and colleagues [27] who compared isolated core exercises with integrated core exercises (front and back squat). They demonstrated greater rectus abdominis activity performing the prone bridge compared the two squat conditions, but similar erector spinae activation. Importantly, the isolated erector spinae exercises were performed as a back extension exercise but on a Swiss ball. This modification reduces the torque of the core (lower moment arm due to the placement of the ball and a more elevated upper body) compared to the present study. Secondly, erector spinae is a prime extensor of the pelvis. Due to the more vertical position of the trunk performing the lunges than squats, greater muscle activation would be expected performing squats compared to the lunge exercise (lower torque) in the present study.

Greater peak core activation in rectus abdominis and erector spinae were observed when performing isolated exercises compared to the three lunge conditions. For oblique externus, the muscle activation performing the isolated core exercise (side bride) was only greater than the stable lunge. These results were partly as hypothesized since the authors expected that the increased torque of the core during lunges with an elastic band would result in similar core muscle activation as the isolated exercises. The increase torque of the core probably resulted in greater erector spinae activation using the elastic bands than the stable and unstable lunges without any external resistance beyond body-weight. Similar findings have been reported previously [15].

Performing the lunges, one foot is elevated before it is lowered to the floor. From the Fig 4, there is one “peak” of core muscle activation—the turnover from eccentric to concentric. The peak involves a deceleration before an acceleration of the body where the core muscle activation increases to maintain trunk position. When lifting the foot, the core muscles’ activations were reduced to almost a minimum compared to peak activation in the push-off (turnover from concentric to eccentric phase) and the start/end of one each repetition (turnover from eccentric to concentric phase). These variations in core muscle activations through a repetition mask the peak activation when only the mean activation is analyzed. Previous comparable studies have analyzed the mean core activation, and this may be the reason why isolated core exercises have demonstrated greater muscle activation than low-load integrated core exercises [27, 33]. In contrast to our findings, studies included high-intensity external load (70% of 1RM) have demonstrated greater activation performing integrated core exercises compared to isolated exercises [29, 30].

Previous studies have demonstrated increased core muscle activation when increasing the resistance [39, 40] and the torque [17, 18, 20]. For example, Sundstrup and colleagues [41] demonstrated greater rectus abdominis activation when performing crunches on a Swiss ball compared to crunches in a training machine. Therefore, it was surprising that the lunges with external loading did not still provide greater muscle activation than the lunges on a stable and unstable surface even though they demonstrated lower activation than the isolated core exercises. One may therefore speculate that an external load of 75N was not sufficient [29, 30]; however, the pilot testing demonstrated that the participants were not able to perform the lunges correctly when a higher force was applied. The present study did not test the glutes or the quadriceps. One could speculate that glutes and quadriceps may have contributed in a greater extent to avoid extension or flexion performing lunges with elastic bands. Further studies should therefore include measurement of these muscle groups.

As hypothesized, similar muscle activation was observed comparing stable vs unstable surface. Similar muscle activation may have been caused by similar load or relative similar stability requirements of the core. Previous studies examining core muscle activation performing lower extremity exercises have used isometric contraction [42, 43] or high-intensity resistance [9]. However, Calatayud et al [16] examined core muscle activation of supine bi-and unilateral plank exercises performed on a stable and an unstable surface (slings). Similar core muscle activation were observed between the conditions [16], which is consistent with the present findings. Still, some previous studies have demonstrated greater core muscle activation when performing exercises on an unstable compared to a stable surface [2, 9]. However, core strengthening exercises were examined (sit-ups) which may explain the contradictory findings to the present study. For studies which have examined multi-joint exercises, the results are not conclusive [11, 27, 33, 42].

Several previous studies have demonstrated possibilities to increase core muscle activation in different core and resistance exercises [16, 25, 44]. However, few studies have compared traditional rehabilitation exercises isolating specific muscle groups with exercises integrating the core [27, 33]. These exercises may mimic daily tasks, prevent low back pain or improve athletic performance in a more functional way [37]. In daily tasks the core muscles must be activated and coordinated to resist forces (i.e. maintaining trunk position stepping down or lifting one bag). When clients perform integrated core exercises, muscle deficits may be observed with experienced trained eyes. The present results demonstrated both advantages and limitations using an integrated approach. Strategies to minimize dysfunction between local and global stabilizers [45] may have greater impact to prevent low back pain than improving strength in the core. For example, less than 25% of maximal voluntary contraction is necessary to stiffen the spinal segments in the core [46, 47]. The use of integrated core training may therefore combine stability, strength and motor control which can lead to improved locomotion in daily movement.

The present study has several limitations. For EMG measurement, there is always an inherent risk of crosstalk between surrounding muscles in addition to methodological concerns with dynamic EMG measurements. Second, all participants were healthy people without low back pain and the results may not necessarily be generalizable to other populations. Thirdly, the present study was an acute study. A training intervention examining the chronic effects may have resulted in a different outcome. Finally, one might also speculate that the large standard deviation and small number of participants might have resulted in a type II error.

In conclusion, isolated core exercises demonstrated greater peak and average core muscle activation compared with the three lunge conditions. Furthermore, no difference in the mean and peak muscle activation were observed between the stable and unstable lunge. Therefore we recommend using isolated isometric core exercises for maximal core muscle activation, but to mimic the specificity and to improve the ability of performing daily tasks or athletic performance, we recommend the use of exercises integrating the core muscles.

## Supporting information

S1 File EMG data. (XLSX)
